# Predicting use of a gait-stabilizing device using a Wii Balance Board

**DOI:** 10.1371/journal.pone.0292548

**Published:** 2023-10-05

**Authors:** Sean M. Mullan, Nicholas J. Evans, Daniel K. Sewell, Shelby L. Francis, Linnea A. Polgreen, Alberto M. Segre, Philip M. Polgreen

**Affiliations:** 1 Department of Computer Science, University of Iowa, Iowa City, Iowa, United States of America; 2 Department of Internal Medicine, University of Iowa, Iowa City, Iowa, United States of America; 3 Department of Biostatistics, University of Iowa, Iowa City, Iowa, United States of America; 4 Department of Pharmacy Practice and Science, University of Iowa, Iowa City, Iowa, United States of America; 5 Department of Epidemiology, University of Iowa, Iowa City, Iowa, United States of America; Opole University of Technology: Politechnika Opolska, POLAND

## Abstract

Gait-stabilizing devices (GSDs) are effective at preventing falls, but people are often reluctant to use them until after experiencing a fall. Inexpensive, convenient, and effective methods for predicting which patients need GSDs could help improve adoption. The purpose of this study was to determine if a Wii Balance Board (WBB) can be used to determine whether or not patients use a GSD. We prospectively recruited participants ages 70–100, some who used GSDs and some who did not. Participants first answered questions from the Modified Vulnerable Elders Survey, and then completed a grip-strength test using a handgrip dynamometer. Finally, they were asked to complete a series of four 30-second balance tests on a WBB in random order: (1) eyes open, feet apart; (2) eyes open, feet together; (3) eyes closed, feet apart; and (4) eyes closed, feet together. The four-test series was repeated a second time in the same random order. The resulting data, represented as 25 features extracted from the questionnaires and the grip test, and data from the eight balance tests, were used to predict a subject’s GSD use using generalized functional linear models based on the Bernoulli distribution. 268 participants were consented; 62 were missing data elements and were removed from analysis; 109 were not GSD users and 97 were GSD users. The use of velocity and acceleration information from the WBB improved upon predictions based solely on grip strength, demographic, and survey variables. The WBB is a convenient, inexpensive, and easy-to-use device that can be used to recommend whether or not patients should be using a GSD.

## Introduction

Falls are the leading cause of injury among older adults [1, 2]. Nearly half of the population over the age of 80 suffers from at least one fall per yea [3]. According to the Centers for Disease Control and Prevention, approximately 27,000 older adults die due to falls, and almost 800,000 people require hospitalization after a fall each year [1]. Fall-related injuries are a major reason for loss of independence among older adults [4, 5], and the fear of falling is detrimental to quality of life for older adults [6].

Several successful programs for preventing falls exist, such as exercise programs [7–9], medication review [7, 10], and interventions to make home environments safer (e.g., installing hand rails, safer bath mats, etc.) [11]. In addition to these strategies, *gait-stabilizing devices* (GSDs) such as canes or walkers may also help prevent falls [12]. GSDs have been shown to be effective particularly in adverse conditions such as the presence of snow or ice [13]. Unfortunately, empirical evidence suggests that most at-risk individuals are not using GSDs when they fall: indeed, many at-risk individuals do not start using such devices until after experiencing a severe fall [14]. Given that canes and walkers are inexpensive and effective fall-prevention measures, encouraging their use has the potential to greatly improve quality of life for at-risk individuals, if only those at risk could be easily identified.

Simple survey-based risk assessment scores are available to help determine the risk of future falls for seniors [15, 16]. Unfortunately, these require trained survey administrators and may be subject to recall bias. In contrast, performative evaluations (e.g., the “Functional Reach” test) require subjects to execute specified tasks [17–19]. While such tests have been empirically validated, they must also be administered by trained personnel and can be too time consuming to be useful in busy clinical settings such as primary care clinics. Fall detection using cameras, smartphones, and accelerometers have been proposed, but further testing is needed [20]. Finally, tests based on specialized technology (e.g., devices with force plates) have also been developed [21–23], but these devices are expensive and impractical when used outside research settings or large clinical centers.

The goal of this project was to build an accurate and inexpensive system to determine if an individual uses GSDs. To do this, we used the output (i.e., the trace of a patient’s shifting center of gravity) from an inexpensive and readily available device, the Wii Balance Board (WBB), to measure postural stability among older adults who use GSDs well as those who do not use GSDs. Then, after combining these data with measurements of grip strength and a few survey questions, we used logistic regression to determine GSD use. The resulting classification system could potentially, in the future, be used to recommend GSD use to new individuals explicitly based on their overall similarity to GSD users in the model. Because people are often reluctant to use a GSD, the ability to quickly and reliably screen non-GSD users whose stability profile aligns more with GSD users identifies a promising target population for a wide range of fall-reduction interventions.

## Materials and methods

### Participants

In our community, we estimated that approximately 10,000 people were eligible for this study. Two hundred and sixty-eight participants between 70 and 100 years of age were recruited: sixty-two had missing data elements and were excluded, but both GSD (i.e., canes, walkers, etc.) users and non-GSD users were included in the final pool. Participants were recruited from multiple retirement communities and nursing homes across the state of Iowa; the Internal Medicine Clinic at the University of Iowa Hospitals and Clinics; a mass email sent to all University of Iowa students, faculty, and staff; and the Seniors Together in Aging Research (STAR) Registry. All participants provided written informed consent. The study was approved by the University of Iowa Institutional Review Board.

### Procedures

The study consisted of one appointment lasting 20 to 30 minutes. Each participant was asked to self-identify as a GSD user (GSD+) or a GSD non-user (GSD-). Participants completed a short questionnaire that consisted of the Vulnerable Elders Survey [24], the participant’s sex, and how many falls and/or near falls they had experienced in the last year. Responses were recorded by a research team member.

Next, participants completed a grip-strength test using a handgrip dynamometer (Jamar, Creative Health Products, Plymouth, MI, USA). Each participant was instructed to sit in an armless chair preferably without leaning back against the chair. With elbows flexed at a 90° angle and not touching the side of the body, participants were then asked to first inhale and then exhale while squeezing the dynamometer as firmly and quickly as possible. Grip strength was measured twice for each hand, alternating between hands to allow for recovery time.

**Fig 1:**
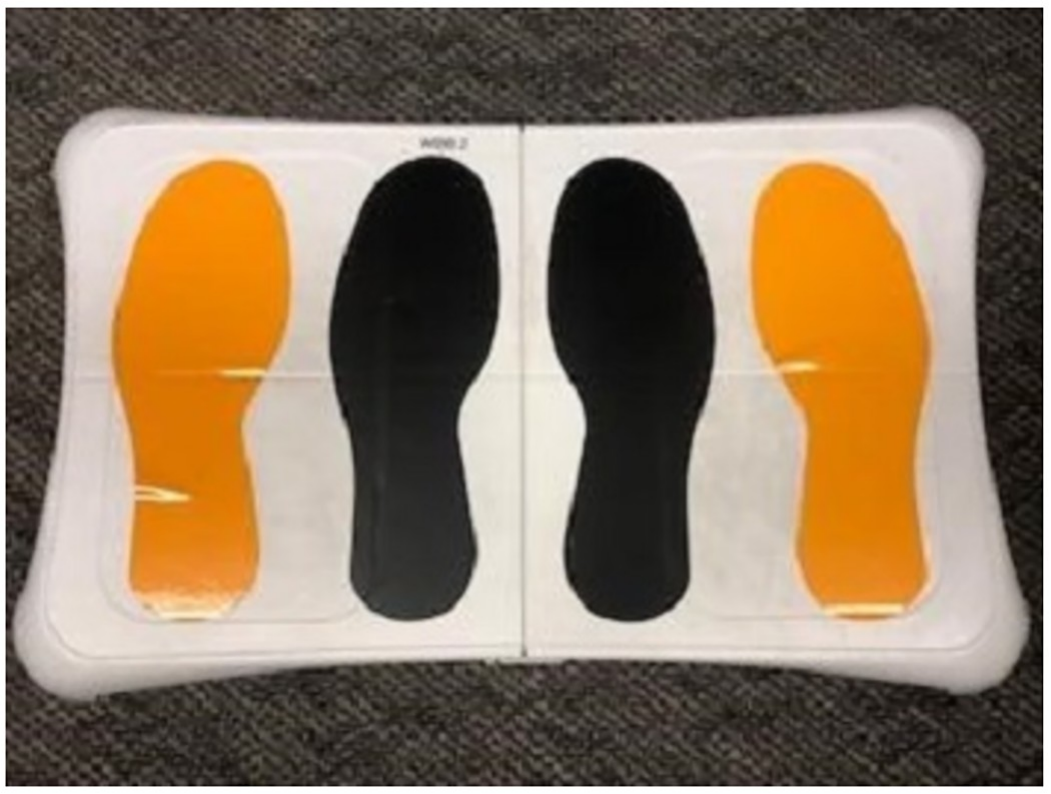
WBB used for four balance trials. When completing a “feet together” trials, participants were asked to stand on the black footprints. When completing a “feet apart” trials, participants were asked to stand on the gold footprints.

**Fig 2:**
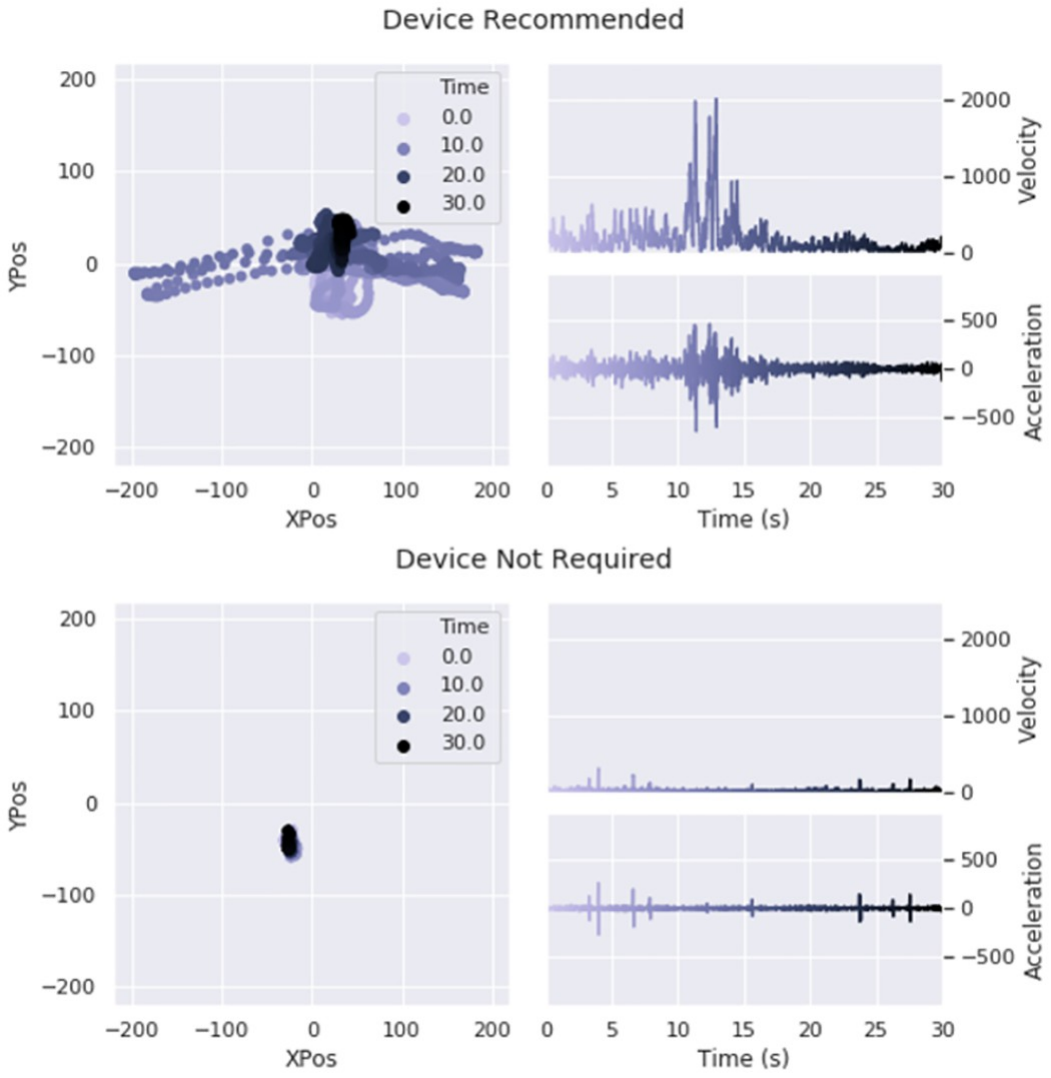
Visualizations of a GSD+ (top) and a GSD- (bottom) individual’s center of balance over the duration of a “eyes closed, feet apart” trial. The left graph of each sample shows the participant’s center of balance over time, and the right graphs show the corresponding velocity and acceleration plotted over time.

Finally, participants were asked to complete a series of four balance tests while standing as still as possible on a WBB. Each test consisted of holding one of four specified poses for 30 seconds: (1) eyes open, feet apart; (2) eyes open, feet together; (3) eyes closed, feet apart; and (4) eyes closed, feet together (see Fig 1). Pose ordering was randomized for each participant. Participants were allowed to rest as needed between balance tests, and all four tests were repeated a second time (in the same initially randomized order). Upon completion of the appointment, participants were mailed a US$5 check for their participation. The research team members were trained to “spot” each participant throughout the balance testing as a precautionary measure. A visualization of each subject’s balance was provided. Fig 2 gives examples of GSD+ and a GSD- individuals.

### Data preparation

Each participant was labeled GSD+ or GSD- depending on how they self-identified: this is the “ground truth” value we are interested in learning to predict. Survey responses were encoded using a Likert scale from 0 (‘Poor’) to 4 (‘Excellent’) or 0 (‘No Difficulty’) to 4 (‘Unable to do’) as appropriate to each individual question, and handgrip scores were measured in kilograms (kgs) of pressure. Together with age and sex, this provided a total of 25 non-WBB features per participant (age, sex, 19 survey responses, and 4 grip strength measures collected over two trials). Seven subjects were missing 4 or fewer values in the demographics, survey, and grip variables. For these subjects we imputed the missing values using predictive mean matching via multivariate imputation by chained equations [25].

Data from the balance tests were encoded as sequences of X and Y center of balance locations in roughly 0.02 second intervals. From these data, we computed the sequences of *velocity* and *acceleration* metrics, where *velocity* is defined as change in X and Y location per timestep, and *acceleration* is defined as the rate of change in velocity per unit of time. To ensure data from different subjects and different trials were comparable, we considered the velocity/acceleration every 1/10^th^ second, so that each test contains 300 (= 30/0·1) velocity measurements and 300 acceleration measurements. For each test within each trial, we created a feature by taking the sum of the square of these 300 velocity measurements and treated this feature as a functional covariate; we treated acceleration similarly. We also aggregated these data across all four tests in trial 1 as well as over all eight tests in both trials. This yielded 20 functional variables per subject (8 tests + aggregation over trial 1 + aggregation over both trials, x2 for both velocity and acceleration).

### Models

We fit generalized functional linear models based on the Bernoulli distribution, using the functional covariates as well as the survey responses and grip strength measurements with the goal of determining the added value of the WBB data. Each predictive model had either zero or one functional covariate, as there were insufficient data to estimate more than one functional covariate at a time.

Model performance was evaluated through 10-fold cross validation, where participants were randomly assorted into 10 like-sized subgroups and the models were trained on either 185 or 186 participants (90%) from 9 of these groups and then tested on the remaining 20 or 21 participants (10%) in the last group. Within each fold, we used backward selection with AIC as the selection criterion on the 25 non-WBB variables. For each functional covariate, we then used functional data analysis (FA) to combine the selected non-WBB variables with the functional variable. FA estimates regression *curves* for functional variables in addition to regression coefficients for non-WBB variables. These regression curves are integrated against the functional variable to determine the predicted probability of needing a GSD. Some subjects were unable to complete any of their balance trials without assistance. Under the logic that such an individual is more likely to need a GSD than even the least stable subjects who were able to complete their balance trials without assistance, we automatically set their predicted value to be GSD+. That is, if a subject were able to complete their balance trials without assistance, we used the generalized functional linear models to predict GSD+/GSD-, and if a subject were not able to complete their balance trials without assistance we predicted GSD+. The out-of-sample predictive performance of these models was evaluated using area under the ROC curve (AUC), accuracy, sensitivity, and specificity. The threshold for making precise predictions from predicted probabilities was set in such a way to ensure a specificity of 0·75 or greater. All analyses were conducted using R version 4.0.4 [26].

## Results

Two-hundred sixty-eight people participated in this study. Twenty-one were removed because of equipment error, 39 subjects were missing survey data, and 2 participants were missing WBB data. Of the remaining 206 participants, 109 self-identified as GSD-, and 97 self-identified as GSD+. Characteristics of the participants can be found in Table 1.

**Table 1:** Participant characteristics based on Gait-Stabilizing-Device (GSD) Group.

	GSD+ (n = 97)	GSD- (n = 109)	p-value
	**Median (IQR)**	**Median (IQR)**	
**Age**	87.0 (81.0, 91.0)	79.0 (74.0, 84.0)	<0.001
**Grip (kg)**			
**Right 1**	16.0 (12.0, 20.0)	24.0 (18.0, 30.5)	<0.001
**Left 1**	14.0 (12.0, 20.0)	21 (16.0, 28.0)	<0.001
**Right 2**	16.0 (12.0, 22.0)	24.0 (18.0, 30.0)	<0.001
**Left 2**	14.0 (12.0, 20.5)	22.0 (16.0, 28.0)	<0.001
	**N (%)**	**N (%)**	
**Sex**			0.557
**Male**	31 (32.0)	40 (36.7)	
**Female**	66 (68.0)	69 (63.3)	
**Near Fall in Past Year**	60 (61.9)	52 (47.7)	0.05
**Fallen in Past Year**	58 (59.8)	31 (28.4)	<0.001
**Location of Falls**			
**Inside Home**	52 (53.6)	19 (17.4)	<0.001
**Outside Home or Yard**	12 (12.4)	14 (12.8)	1
**In Community**	9 (9.3)	8 (7.3)	0.623
**Difficulty (Yes)**			
**Shopping for Personal Items**	15 (15.5)	1 (0.9)	<0.001
**Managing Money**	4 (4.1)	0 (0.0)	0.048
**Walking Across the Room (using GSD OK)**	6 (6.2)	2 (1.8)	0.152
**Doing Light Housework (washing dishes)**	8 (8.2)	0 (0.0)	0.002
**Bathing or Showering**	32 (33.0)	1 (0.9)	<0.001
**Health Rating Compared to Peers (0 = Poor; 1 = Fair; 2 = Good; 3 = Very good; 4 = Excellent)**			<0.001
**0**	1 (1.0)	2 (1.8)	
**1**	26 (26.8)	3 (2.8)	
**2**	45 (46.4)	31 (28.4)	
**3**	18 (18.6)	48 (44.0)	
**4**	7 (7.2)	25 (22.9)	
**Difficulty (0 = None; 1 = Little; 2 = Some; 3 = Much; 4 = Unable to do)**			
**Stooping, Crouching, Kneeling**			<0.001
**0**	7 (7.2)	46 (42.2)	
**1**	13 (13.4)	32 (29.4)	
**2**	28 (28.9)	22 (20.2)	
**3**	16 (16.5)	7 (6.4)	
**4**	33 (34.0)	2 (1.8)	
**Lifting/Carrying 10 Pounds**			<0.001
**0**	53 (54.6)	92 (84.4)	
**1**	18 (18.6)	8 (7.3)	
**2**	11 (11.3)	8 (7.3)	
**3**	4 (4.1)	1 (0.9)	
**4**	11 (11.3)	0 (0.0)	
**Reaching/Extending Arms Overhead**			0.001
**0**	69 (71.1)	100 (91.7)	
**1**	12 (12.4)	5 (4.6)	
**2**	10 (10.3)	4 (3.7)	
**3**	3 (3.1)	0 (0.0)	
**4**	3 (3.1)	0 (0.0)	
**Writing/Handling/Grasping Small Objects**			<0.001
**0**	59 (60.8)	96 (88.1)	
**1**	21 (21.6)	10 (9.2)	
**2**	12 (12.4)	3 (2.8)	
**3**	5 (5.2)	0 (0.0)	
**4**	0 (0)	0 (0.0)	
**Walking a Quarter of a Mile**			<0.001
**0**	43 (44.3)	91 (83.5)	
**1**	16 (16.5)	9 (8.3)	
**2**	9 (9.3)	7 (6.4)	
**3**	10 (10.3)	1 (0.9)	
**4**	19 (19.6)	1 (0.9)	
**Heavy Housework (floors, windows)**			<0.001
**0**	12 (12.4)	66 (62.3)	
**1**	17 (17.5)	20 (18.9)	
**2**	14 (14.4)	9 (8.5)	
**3**	8 (8.2)	5 (4.7)	
**4**	46 (47.4)	6 (5.7)	

P-values computed using Fisher’s exact test (categorical variables) or Kruskal Wallis (continuous variables).

Table 2 shows how many folds (i.e., training data sets) included each non-WBB variable, as well as which variables were included when performing backward selection on the entire data set. The out-of-sample prediction performance is given in Table 3. All WBB functional variables helped to improve the predictive performance. The best performing model corresponded to the inclusion of the velocity aggregated over the first four tests. However, even using a single test such as “eyes closed, feet together” made meaningful improvements in the predictive performance compared to only using the non-WBB covariates.

**Table 2:** Number of folds (out of 10) which led to the inclusion of each non-WBB covariate when performing backward model selection.

Variable	# Times included in model selection process
**Age**	10*
**Sex**	10*
**Grip (kg)**	
**Right / Trial 1**	10*
**Right / Trial 2**	0
**Left / Trial 1**	1
**Left / Trial 2**	0
**Health Rating Compared to Peers (0–4)**	7*
**Difficulty Rating (0–4)**	
**Stooping, Crouching, Kneeling**	10*
**Lifting/Carrying 10 Pounds**	1
**Reaching/Extending Arms Overhead**	10*
**Writing/Handling/Grasping Small Objects**	4
**Walking a Quarter of a Mile**	9*
**Heavy Housework (floors, windows)**	2
**Has Difficulty (Y/N)**	
**Shopping for Personal Items**	1
**Managing Money**	1
**Walking Across the Room (using AD OK)**	0
**Doing Light Housework (washing dishes)**	0
**Bathing or Showering**	10*
**Fallen in Past Year (Y/N)**	1
**Number of Falls (if fallen)**	8*
**Location of Falls (Y/N)**	
**Inside Home**	0
**Outside Home or Yard**	9*
**In Community**	0
**Nearly Fallen in Past Year (Y/N)**	6
**Number of Near-Falls**	1

* Included when performing model selection on the full data set

**Table 3:** Out-of-sample prediction performance for each of the 21 models.

	Trial 1 Only				Trials 1 and 2			
	Specificity	Sensitivity	Accuracy	AUC	Specificity	Sensitivity	Accuracy	AUC
**Velocity**								
**Open/ Together**	0·752	0·887	0·816	0·884	0·752	0·887	0·816	0·884
**Open/Apart**	0·752	0·897	0·820	0·885	0·752	0·897	0·820	0·885
**Closed/Together**	0·752	0·887	0·816	0·896	0·752	0·887	0·816	0·896
**Closed/Apart**	0·752	0·866	0·806	0·880	0·752	0·866	0·806	0·880
**All**	0·752	0·918	0·830	0·899	0·752	0·866	0·806	0·881
**Acceleration**								
**Open/Together**	0·752	0·845	0·796	0·878	0·752	0·845	0·796	0·878
**Open/Apart**	0·752	0·856	0·801	0·883	0·752	0·856	0·801	0·883
**Closed/Together**	0·752	0·876	0·811	0·886	0·752	0·876	0·811	0·886
**Closed/Apart**	0·752	0·876	0·811	0·877	0·752	0·876	0·811	0·877
**All**	0·752	0·835	0·791	0·893	0·752	0·876	0·811	0·883
**Non-WBB Variables**								
	0·752	0·804	0·777	0·873				

**Fig 3:**
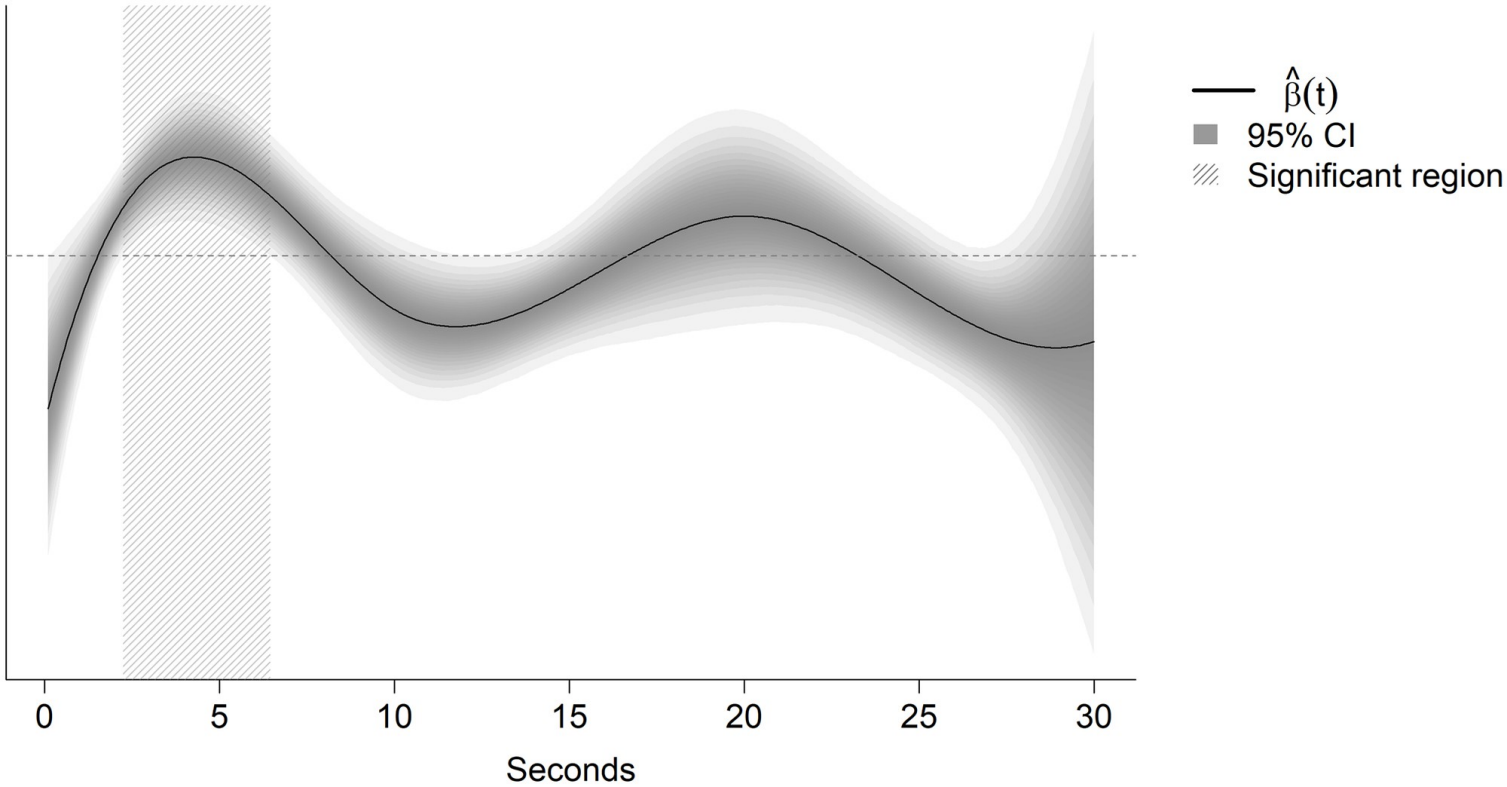
Regression curve (black) from the functional data analysis and the 95% confidence bands (gray), using the velocity aggregated over the four tests of trial 1 as the functional covariate, where the horizontal axis is the time since beginning the test. The dashed horizonal line is located at zero: locations where the curve is above this line represents time periods where large values of velocity increase the predicted probability of needing a GSD. The region statistically significantly different from zero is from 2.25 sec to 6.44 sec. (hatched region), indicating the period during when large velocities would predict a high probability of needing a GSD.

Inspecting the FA regression curves provides insight into the time period during which the balance board data helps to predict GSD. Fig 3 shows the regression curves and their 95% confidence bands for the best predictive model, i.e., the model with non-WBB covariates and the velocity aggregated over the four tests in trial 1. This curve shows that the balance board data statistically significantly improved predictions from ~2.25 sec to ~6.50 sec.

## Discussion

Our results demonstrate that we can accurately identify people who currently use a GSD based on their answers to a few simple questions and their performance on a brief balance test using only inexpensive equipment. Given the accuracy of our results, our system could be used to recommend the use of GSDs to people who are currently not using one but that would likely benefit from a GSD and other fall-prevention interventions. Our system also produces a convincing comparative visualization of each participant’s postural stability, which can be used to better communicate a subject’s relative instability, possibly helping to overcome device- adoption barriers.

The risk for falls is often multi-factorial. Our approach quickly measures two important risk factors including postural stability (via the WBB) and grip strength (via a simple hand-grip dynamometer). Our best performing model included velocity aggregated over the first 4 tests in trial 1. Given the ease of our approach, it provides a few distinct advantages. First, our approach could be used quickly and efficiently (about 5 minutes per patient) in a clinical setting without the use of specialized or expensive equipment (WBB are out of production but used WBB can easily be purchased for approximately $25). Also, the WBB is small, portable and could be placed next to a scale. It does not need personnel with specific training or a large, designated space both of which may be difficult to obtain in many clinical settings. Second, our approach produces an objective measurement. While the Get-Up-and-Go test, as well as other functional tests geared toward older adults/seniors [27], have been validated, and used in machine-learning models [28–30], because these tests are based on an observer with a timer, there may be some subjectivity associated with interpretation of observations that may vary by observer, especially in busy clinical settings. Third, the data we collect measuring postural stability can be readily reduced to an informative graphical visualization so that patients can easily compare their results to population averages. While not an objective of our study, participants and family members frequently asked to see the trace after the study was completed and found the trace helpful, allowing them to easily interpret the results. Future work will need to determine if and to what extent such information influences patients enough to change behavior (e.g., increase the likelihood that a patient would agree to acquire and use a GSD.)

Despite the effectiveness of GSDs, possession of a GSD does not ensure its use. Furthermore, it is difficult to monitor adherence to such devices. Indeed, people who do fall and own a GSD are often not using it at the time of their fall [14]. In recent years, a growing body of qualitative research has provided some insight into factors that may influence people’s willingness to use GSDs [31–33]. These studies suggest that the use of GSDs negatively affect self-identity and impose many cognitive, behavioral, and pragmatic adaptations. More specifically, personal beliefs concerning physical, psychological and social consequences of device use and the ease or difficulty anticipated in successfully incorporating the device into one’s daily activities appear to be the key factors influencing the decisions to accept or reject GSDs [31–33].

Our approach, with its clear visualization of postural stability could be used to help encourage GSD use among people who have such a device, but who do not use it regularly. Our approach could also be used to screen patients who would likely benefit from a wide range of other fall-prevention efforts. In addition, given the granularity of our postural stability measurements, we might be able to build new models in future applications to determine if we can detect improvements in postural stability. For example, while our FA model was successful at prediction, the number of subjects in our study was sufficiently small as to preclude the use of multiple functional covariates in the same model. Indeed, while we built a similar FA model using squared acceleration rather than velocity with very similar results, there were insufficient data to include both velocity and acceleration functional covariates simultaneously. We believe it likely that given a larger number of subjects we would be able to train FA models using more nuanced functional covariates, thereby enabling us to build a more accurate and comprehensive picture of patients’ postural stability.

The WBB has previously been used to assess postural stability, a well described risk factor for falls. In general, the WBB compares favorably to research-grade force plates, which are much more expensive [34]. WBBs have also been used to predict future fall risk [35, 36], and complement the Get-Up-and-Go test [37]. However, these approaches have been mostly confined to research settings and have not been widely generalized to busy clinical settings. Given the initial popularity of the device in gaming systems, and because Nintendo is no longer supporting the use of the WBB with its newer gaming systems, there will be a steady and inexpensive supply of these devices and systems as people upgrade to newer platforms for years to come.

Our results are subject to several limitations. First, our results are based on a limited sample of only 206 participants, albeit one with a relatively balanced training set (49% of subjects used GSDs). While our population is limited, in general, increasing our sample should increase and not decrease our model’s performance. Second, it may be that some of the people in our study were assigned to either the GSD+ or GSD- group incorrectly. Third, recommendations alone may not be convincing enough to change user behavior and increase GSD use: future work will be needed to show if our more objective approach can be used to change adherence to GSDs. Fourth, the signals from the WBB are noisy, but we were still able to predict GSD use. Finally, for the purposes of this initial work, we did not distinguish between different types of GSDs (i.e., canes vs. walkers). Future work including larger samples and ways to include patient and family preference are needed. In conclusion, despite these limitations, we demonstrate a convenient, inexpensive and easy-to-use approach for identifying patients who are currently using a GSD.

## References

[1] Bergen G, Stevens MR, Burns ER. Falls and fall injuries among adults aged≥ 65 years—United States, 2014. *Morbidity and Mortality Weekly Report*. 2016;65(37):993–998. 27656914 10.15585/mmwr.mm6537a2

[2] Stevens JA, Mack KA, Paulozzi LJ, Ballesteros MF. Self-reported falls and fall-related injuries among persons aged≥ 65 years–United States, 2006. *Journal of safety research*. 2008;39(3):345–349. 18571577 10.1016/j.jsr.2008.05.002

[3] Tinetti ME, Speechley M, Ginter SF. Risk factors for falls among elderly persons living in the community. *New England journal of medicine*. 1988;319(26):1701–1707. doi: 10.1056/NEJM198812293192604 3205267

[4] Tinetti ME, Williams CS. The effect of falls and fall injuries on functioning in community-dwelling older persons. *The Journals of Gerontology Series A*: *Biological Sciences and Medical Sciences*. 1998;53(2):M112–M119. doi: 10.1093/gerona/53a.2.m112 9520917

[5] Tinetti ME, Williams CS. Falls, injuries due to falls, and the risk of admission to a nursing home. *New England journal of medicine*. 1997;337(18):1279–1284. doi: 10.1056/NEJM199710303371806 9345078

[6] Whitehouse JD, Friedman ND, Kirkland KB, Richardson WJ, Sexton DJ. The impact of surgical-site infections following orthopedic surgery at a community hospital and a university hospital adverse quality of life, excess length of stay, and extra cost. *Infection Control & Hospital Epidemiology*. 2002;23(4):183–189. 12002232 10.1086/502033

[7] Gillespie LD, Robertson MC, Gillespie WJ, et al. Interventions for preventing falls in older people living in the community. *Cochrane database of systematic reviews*. 2012;(9). doi: 10.1002/14651858.CD007146.pub3 22972103 PMC8095069

[8] Okubo Y, Schoene D, Lord SR. Step training improves reaction time, gait and balance and reduces falls in older people: a systematic review and meta-analysis. *British journal of sports medicine*. 2017;51(7):586–593. doi: 10.1136/bjsports-2015-095452 26746905

[9] Yamada M, Tanaka B, Nagai K, Aoyama T, Ichihashi N. Trail-walking exercise and fall risk factors in community-dwelling older adults: preliminary results of a randomized controlled trial. *Journal of the American Geriatrics Society*. 2010;58(10):1946–1951. doi: 10.1111/j.1532-5415.2010.03059.x 20831723

[10] Pit SW, Byles JE, Henry DA, Holt L, Hansen V, Bowman DA. A Quality Use of Medicines program for general practitioners and older people: a cluster randomised controlled trial. *Medical Journal of Australia*. 2007;187(1):23–30. doi: 10.5694/j.1326-5377.2007.tb01110.x 17605699

[11] Keall MD, Pierse N, Howden-Chapman P, et al. Home modifications to reduce injuries from falls in the home injury prevention intervention (HIPI) study: a cluster-randomised controlled trial. *The Lancet*. 2015;385(9964):231–238. doi: 10.1016/S0140-6736(14)61006-0 25255696

[12] Gell NM, Wallace RB, LaCroix AZ, Mroz TM, Patel KV. Mobility device use in older adults and incidence of falls and worry about falling: Findings from the 2011–2012 national health and aging trends study. *Journal of the American Geriatrics Society*. 2015;63(5):853–859. doi: 10.1111/jgs.13393 25953070 PMC4439269

[13] McKiernan FE. A simple gait-stabilizing device reduces outdoor falls and nonserious injurious falls in fall-prone older people during the winter. *Journal of the American Geriatrics Society*. 2005;53(6):943–947. doi: 10.1111/j.1532-5415.2005.53302.x 15935015

[14] Luz C, Bush T, Shen X. Do canes or walkers make any difference? Nonuse and fall injuries. *The Gerontologist*. 2017;57(2):211–218. doi: 10.1093/geront/gnv096 26209797

[15] Tromp A, Pluijm S, Smit J, Deeg D, Bouter L, Lips P. Fall-risk screening test: a prospective study on predictors for falls in community-dwelling elderly. *Journal of clinical epidemiology*. 2001;54(8):837–844. doi: 10.1016/s0895-4356(01)00349-3 11470394

[16] Palumbo P, Klenk J, Cattelani L, et al. Predictive performance of a fall risk assessment tool for community-dwelling older people (FRAT-up) in 4 European cohorts. *Journal of the American Medical Directors Association*. 2016;17(12):1106–1113. doi: 10.1016/j.jamda.2016.07.015 27594522 PMC6136246

[17] Duncan PW, Studenski S, Chandler J, Prescott B. Functional reach: predictive validity in a sample of elderly male veterans. *Journal of gerontology*. 1992;47(3):M93–M98. doi: 10.1093/geronj/47.3.m93 1573190

[18] De Rekeneire N, Visser M, Peila R, et al. Is a fall just a fall: correlates of falling in healthy older persons. The Health, Aging and Body Composition Study. *Journal of the American Geriatrics Society*. 2003;51(6):841–846. doi: 10.1046/j.1365-2389.2003.51267.x 12757573

[19] Verghese J, Buschke H, Viola L, et al. Validity of divided attention tasks in predicting falls in older individuals: a preliminary study. *Journal of the American Geriatrics Society*. 2002;50(9):1572–1576. doi: 10.1046/j.1532-5415.2002.50415.x 12383157

[20] Lapierre N, Neubauer N, Miguel-Cruz A, Rios Rincon A, Liu L, Rousseau J. The state of knowledge on technologies and their use for fall detection: A scoping review. *International Journal of Medical Informatics*. 2018/03/01/ 2018;111:58–71. doi: 10.1016/j.ijmedinf.2017.12.015 29425635

[21] Pajala S, Era P, Koskenvuo M, Kaprio J, Törmäkangas T, Rantanen T. Force platform balance measures as predictors of indoor and outdoor falls in community-dwelling women aged 63–76 years. *The Journals of Gerontology Series A*: *Biological Sciences and Medical Sciences*. 2008;63(2):171–178. doi: 10.1093/gerona/63.2.171 18314453

[22] Swanenburg J, de Bruin ED, Uebelhart D, Mulder T. Falls prediction in elderly people: a 1-year prospective study. *Gait & posture*. 2010;31(3):317–321. doi: 10.1016/j.gaitpost.2009.11.013 20047833

[23] Hewson DJ, Singh NK, Snoussi H, Duchene J. Classification of elderly as fallers and non-fallers using centre of pressure velocity. IEEE; 2010:3678–3681. 10.1109/IEMBS.2010.5627649 21097047

[24] Saliba D, Elliott M, Rubenstein LZ, et al. The Vulnerable Elders Survey: a tool for identifying vulnerable older people in the community. *Journal of the American Geriatrics Society*. 2001;49(12):1691–1699. doi: 10.1046/j.1532-5415.2001.49281.x 11844005

[25] Van Buuren S, Groothuis-Oudshoorn K. mice: Multivariate imputation by chained equations in R. *Journal of statistical software*. 2011;45(1):1–67.

[26] Core R. TEAM, 2022. R: A language and environment for statistical computing. R Foundation for Statistical Computing, Vienna, Austria. Online: https://www.r-project.org. 2022;

[27] Rikli RE, Jones CJ. *Senior fitness test manual*. Human kinetics; 2013.

[28] Makino K, Lee S, Bae S, et al. Simplified decision-tree algorithm to predict falls for community-dwelling older adults. *Journal of clinical medicine*. 2021;10(21):5184. doi: 10.3390/jcm10215184 34768703 PMC8585075

[29] Speiser JL, Callahan KE, Houston DK, et al. Machine learning in aging: an example of developing prediction models for serious fall injury in older adults. *The Journals of Gerontology*: *Series A*. 2021;76(4):647–654. doi: 10.1093/gerona/glaa138 32498077 PMC8011704

[30] Hua A, Quicksall Z, Di C, et al. Accelerometer-based predictive models of fall risk in older women: a pilot study. *NPJ digital medicine*. 2018;1(1):1–8. doi: 10.1038/s41746-018-0033-5 31304307 PMC6550179

[31] Aminzadeh F, Edwards N. Exploring seniors’ views on the use of assistive devices in fall prevention. *Public health nursing*. 1998;15(4):297–304. doi: 10.1111/j.1525-1446.1998.tb00353.x 9682623

[32] Bates PS, Spencer JC, Young ME, Rintala DH. Assistive technology and the newly disabled adult: adaptation to wheelchair use. *American Journal of Occupational Therapy*. 1993;47(11):1014–1021. doi: 10.5014/ajot.47.11.1014 8279496

[33] Scheer J, Luborsky ML. The cultural context of polio biographies. SLACK Incorporated Thorofare, NJ; 1991. 10.3928/0147-7447-19911101-05 PMC4241342 1758785

[34] Clark RA, Mentiplay BF, Pua Y-H, Bower KJ. Reliability and validity of the Wii Balance Board for assessment of standing balance: A systematic review. *Gait & posture*. 2018;61:40–54. doi: 10.1016/j.gaitpost.2017.12.022 29304510

[35] Johansson J, Nordström A, Gustafson Y, Westling G, Nordström P. Increased postural sway during quiet stance as a risk factor for prospective falls in community-dwelling elderly individuals. *Age and ageing*. 2017;46(6):964–970. doi: 10.1093/ageing/afx083 28531243

[36] Howcroft J, Lemaire ED, Kofman J, McIlroy WE. Elderly fall risk prediction using static posturography. *PLoS one*. 2017;12(2):e0172398. doi: 10.1371/journal.pone.0172398 28222191 PMC5319679

[37] Kwok B-C, Clark RA, Pua Y-H. Novel use of the Wii Balance Board to prospectively predict falls in community-dwelling older adults. *Clinical biomechanics*. 2015;30(5):481–484. doi: 10.1016/j.clinbiomech.2015.03.006 25796535

